# Exploring patient perspectives on a new task-shared behavioral health role in Washington State

**DOI:** 10.1371/journal.pmen.0000606

**Published:** 2026-06-29

**Authors:** Alexandra L. Rose, Olga Vitruk, Brenna N. Renn, Lydia Chwastiak, William P. O’Connell, Anna Ratzliff

**Affiliations:** 1 University of Washington Department of Psychiatry and Behavioral Sciences, Seattle, Washington, United States of America; 2 University of Nevada, Las Vegas Department of Psychology, Las Vegas, Nevada, United States of America; 3 University of Washington Department of Global Health, Seattle, Washington, United States of America; National University of Singapore, SINGAPORE

## Abstract

Mental health provider shortages lead to substantial treatment gaps for mental health services worldwide, including throughout the United States. Task-sharing, in which providers with no or limited prior training in mental health care are trained to deliver psychological interventions, is a leading strategy to increase capacity, yet it has been under-utilized in the US to date. Recent legislative changes are starting to enable opportunities for task-sharing mental healthcare in the US. A leading example is Washington State’s introduction of the Behavioral Health Support Specialist (BHSS), a supervised bachelors-level mental health provider role. However, little is known about patient perspectives on task-sharing mental health care within US settings. Our study engaged potential future patients at two integrated care clinics (*n* = 40) using mixed methods data collection (semi-structured individual interviews and brief structured surveys adapted from the acceptability and appropriateness sub-scales of the Mental Health Implementation Science Tools) to explore potential patient perspectives on the perceived acceptability and appropriateness of these reforms in the US. We identified three main qualitative themes: (1) Overall, potential patients reported that they perceived task-shared mental health care to be acceptable; (2) Potential patients reported familiarity with task-sharing from other clinical and community settings; and (3) Potential patients shared hesitations and questions that are important for implementers and educators to address. Quantitatively, most participants reported moderate to strong support for receiving care from a task-shared mental health provider. Given that legislative reform expanding care delivery via these roles is already well underway, our findings can help support successful implementation of a bachelor’s level mental health provider role. Though the scope of this bachelor’s level role is currently limited to Washington, other states are exploring introducing similar roles to their mental health workforce, so these findings may be applicable nationwide.

## Introduction

In 2024, 29.5 million Americans with a mental health condition did not receive any mental health treatment [1]. Mental health provider shortages [2–4] are a key driver of this treatment gap, which is even more substantial outside of the United States [2,5–7]. In the United States, federally designated Mental Health Care Health Professional Shortage Areas (HPSAs), defined by the ratio of mental health professionals relative to the population, were present in every US state [8].

Task-sharing, in which healthcare providers with little or no prior mental health background are trained to deliver psychological interventions under supervision, is a leading strategy to address this global shortage of mental health providers and expand access to mental health services [9–12]. Task-sharing models were initially developed within low-resource health systems to address shortages of HIV providers in the early 2000s [13], and have more recently expanded to other health conditions, including mental health care [14]. Task-sharing mental health care is now widely established in some countries, such as in the United Kingdom through the Improving Access to Psychological Therapies (IAPT)/Talking Therapies program [15], which was used by 1.26 million people in 2023 [16]. In the US, task-sharing is well established in the treatment of medical disorders, such as task-sharing between primary care physicians and medical assistants in primary care settings [17]. Peer support specialists outside the health system have increasingly become involved in task-sharing psychosocial interventions in community-based settings [18–22]. Using task-sharing to introduce more providers into the mental healthcare system is anticipated to expand access to mental healthcare by introducing more staff to the system and allowing the more limited group of advanced mental health professionals to focus their expertise on more complex cases [23]. However, despite well-described provider shortages and mental health care access issues, task-sharing mental health services within the US health system has been limited to date due to governmental regulation and payor requirements that have limited mental health clinical service delivery to master’s level providers [24]. Where bachelor’s-level staff have been involved in mental health service delivery in the US it has largely been in mental health case management, rather than treatment delivery roles [25].

State governments are now beginning to change legislation, credentialling requirements, and billing codes to enable mental health services provided by bachelor’s level and other task-shared providers in US healthcare, including in Washington (WA), Oregon, and Nevada [26–29]**.** In 2023, WA introduced the Behavioral Health Support Specialist (BHSS) [30]. The WA Department of Health (DOH) specified that the BHSS may work under supervision delivering evidence-based psychological interventions (e.g., behavioral activation, motivational interviewing) in any setting offering mental health and behavioral health services in WA to maximize the broad utility of the role [31]. Due to the prevalence of common mental disorders and the relative brevity of BHSS training they are expected to work primarily with patients with mild to moderate depression and anxiety in outpatient settings offering behavioral health services (e.g., primary care mental health, community mental health agencies) [32]. As of 2025, the first BHSSs have graduated from local educational programs that were modified to meet these new requirements, are being credentialled by the DOH and beginning employment as clinicians. It is estimated that the first 50 students will have graduated and 40 BHSSs will be credentialed and employed by 2026 [33]. BHSSs work within a stepped care system [34]; in addition to being supervised by licensed specialists while providing interventions, they refer patients “upward” or “outward” to more specialized providers if their initial provision of low-intensity interventions is not sufficient.

Though formative work was conducted to articulate the BHSS role in WA and adapt local educational programs to enable training [35,36], and more recent studies have started to disseminate lessons from implementation [37], perspectives of potential patients have yet to be solicited about this reform. While task-shared mental health has typically had good acceptability in other settings [38,39], understanding US patient perspectives on receiving mental health care from providers without graduate training is critical to addressing any patient-level barriers to the introduction of these services in the US. Patient engagement and buy-in impacts the uptake and outcomes of health care reform [40]. Though the scope of the BHSS is limited to WA at this time, other states are actively exploring or planning for similar task-shared mental health roles [26–28,41–44], therefore these findings may be relevant throughout the country.

## Materials and methods

### Ethics statement

Approval for this study was provided by the University of Washington Human Subjects Division (STUDY00021389). Participants provided verbal consent for sharing their perspectives and written consent for extraction of data from their medical records.

### Study design and setting

This mixed method study is reported in alignment with the Consolidated Criteria for Reporting Qualitative studies (COREQ) [45] and the Good Reporting of A Mixed Methods Study (GRAMMS) [46] checklists. Please see S1 File and S2 File. Data collection spanned March 17th to June 20^th^, 2025. Due to the emerging nature of the BHSS at that time (i.e., no BHSSs had yet graduated from their training programs), we sampled potential patients that, based on their clinical characteristics, may be likely to receive care from a BHSS in the future at clinics that may be likely to employ a BHSS. In alignment with the Washington State Department of Health and developers of the BHSS role, we defined clinics that may be likely to employ a BHSS in the future as those providing mental health care integrated within primary care settings and managing lengthy waitlists for mental health services.

### Participants and sampling

We planned to sample 40 participants, 20 each from two primary care clinics with integrated mental health services in King County, based on the expected number to reach theoretical saturation from the prior experience of our team and the qualitative methods literature [47]. First, we extracted patients who attended a medical visit with a diagnosis of mild or moderate depression or anxiety in the past 12 months from the electronic medical records of the two clinics. As approved by the UW Human Subjects Division, patients with documentation of cognitive impairment in the medical record were excluded from extraction. Using a random number generator, we then randomly sampled and contacted possible participants by phone with information about the study and to complete the consenting process.

### Data collection

All participants who completed the consenting process continued with the study. First, each participant completed a semi-structured individual interview (approximately 30 minutes) that explored perceived acceptability and appropriateness of task-shared mental health care to participants. The participants had no prior contact with the interviewers aside from screening. Interviews were conducted by ALR, a female postdoctoral researcher with a PhD, and OV, a female research coordinator with a Master of Public Health. Both had previously done research on mental health services in WA and did not share any information about themselves with the participants. Interview guides first asked participants about their understanding of and interest in mental health provider training backgrounds and cooperation with other providers. Next, since participants were unlikely to have had prior contact or familiarity with task-shared mental health providers, interview guides presented two vignettes of task-shared mental health care to participants before asking for feedback on acceptability and appropriateness of these roles. In alignment with the Proctor et al., definitions, acceptability was defined as the participants’ perception that the BHSS role was agreeable, palatable, or satisfactory, and appropriateness was defined as the participants’ perceived fit, relevance, or compatibility of this new role for their given clinic setting and their mental health needs [48]. Lastly, interviews asked about perceived advantages and disadvantages of receiving care from this type of provider. Interviews were conducted via Zoom and audio-recorded, then transcribed. Please see S3 File. Thirty-eight participants completed the interview with a usable audio-recording; one participant did not finish the interview, and the recording was not sufficiently audible on one completed interview.

After completing the interview, participants were asked to complete an adapted version of the acceptability and appropriateness sub-scales of the Mental Health Implementation Science Tools (mhIST) [49] sent to them via REDCap [50]. Prompts included: “Do you think a bachelor’s level provider would have the abilities to address your mental health needs?” and “Do you think bachelor’s level providers are appropriate to be helping people with problems like yours?” Response options ranged from 1 = *Not at all* to 4 = *A lot.* Participants could also select “Don’t know.” Please see S4 File. Thirty-nine participants returned the survey.

### Data analysis

After transcription, interviews were imported into Dedoose qualitative software [51]. ALR and OV then independently coded the interviews using a hybrid deductive-inductive coding framework [51,52], in which the initial codebook was based on the interview guides and additional codes were added through open coding. Hybrid deductive-inductive approaches are appropriate when answering a targeted research question (e.g., what is the acceptability of a task-shared mental health provider to participants?) without missing important findings that may fall outside of the interview guide (e.g., relevant findings related more broadly to mental health care in the United States). The coders then identified key themes through iterative discussion of codes.

Descriptive statistics (frequency and proportion) were calculated for survey responses. Lastly, qualitative and quantitative data sources were integrated to examine the convergence and divergence of potential patient perspectives using an integrated visual joint display table [53]. Comparison in this way helps strengthen findings when there is convergence between methods, and identify areas that lack clarity when there is divergence [54].

## Results

Participants (*N* = 40) were 47.5% female, 45% male, and 7.5% other sex. They were primarily White (67.5%) and on Medicaid or Medicare (67.5%). Around half (52.5%) were included in our sample based on diagnosis with a depressive disorder; the remainder were included for diagnosis with an anxiety disorder or with both anxiety and depression.

### Qualitative themes

Overall, participants described that they were not familiar with mental health provider credentials and were open to receiving care from a task-shared mental health provider. Three key themes were identified relating to their openness to the new role.

#### Theme 1: Appropriateness and acceptability of task-shared mental health care.

Most participants expressed the appropriateness of receiving mental health care from a bachelor’s level task-shared provider when the provider’s scope matched their current needs. Many were able to differentiate times throughout their lives when a task-shared provider would be able to benefit them versus times when a more advanced provider would have been a better fit:

“In like the spot where I am now, I would say [working with a bachelor’s-level provider is] a lot more appropriate because I have done a lot of the work, and I know how to cope with my feelings a lot more and everything. So I think in in my case now, that would be more appropriate. But you know, 8 years ago [when my mother was dying] it wouldn’t have been as appropriate”

- Other gender, 31

This view of the appropriateness of task-sharing was grounded in perceptions by most that task-sharing would expand access to mental health care, namely through the advantage of shorter wait times to see a therapist and greater quantity and diversification of mental health options. One participant explained why enabling patients to see a therapist sooner rather than later is so important, as delayed patient appointments may be detrimental to well-being whereas earlier treatment may prevent later acuity:

“Get care earlier in the depression. And whatever problem I’m struggling with. Start at some at a lower level of care, and see if that’ll kind of nip it in the bud versus sitting with the depression and letting it get worse and worse until you know, I really do need help…or things are gonna go badly…”

- Female, 70

Participants also recognized that this role, situated within a stepped care model, would improve efficiency of the mental health care system by allowing more advanced providers to take on more complex cases. One participant explained this:

“Well, I would hope that there would be more of of those kinds of [bachelor’s-level] providers, thus giving more time and freedom to perhaps more qualified peoples to take on more demanding cases from people who need it more than I would…like I said that there would be more people. I mean, it’s it’s less of a time sink to get just a bachelor’s degree in this. So, having more therapists readily available, can tackle a larger swath of people.”

- Other gender, 26

A number of participants shared that the team element inherent to a supervised task-shared provider supported acceptability. One participant explained how they would appreciate both the task-shared provider and supervisor reviewing cases in this model, while mental health care is historically provided by a siloed provider:

“I do abstractly think that more insight is generated by more than one person. The facts of a case. I think that I mean, I’m a software engineer by profession. And I know that when working on projects, it’s working in pairs is much more efficient than working alone. And so, if the theoretical bachelor’s provider is working in tandem with the supervisor to consider the case, I think that would actually be an advantage than just the supervisor considering the case by themselves.”

- Male, 58

Many participants shared impressions that task-shared mental health providers could be less burnt out and have access to newer, more up-to-date mental health information than traditional providers.

“Another cool thing is because it’s like a new position. And like a, you know, a new whole new idea. I feel like they would probably come in all gung ho. And excited with a lot of energy. They wouldn’t be burnt out…Sometimes you get a doctor, and they’re just like you can tell they’ve been there forever. And then, just, you know, are going through the motions.”

- Female, 46

Further supporting acceptability, several participants believed that task-shared bachelor’s level mental health providers could be more culturally and economically diverse and bring more lived experience to their roles than traditional providers. One participant explained how they imagined the backgrounds of these new providers could be more similar to that of their clients:

“Okay, this is complicated. one of the things I really like about what you are describing Is that you know, there’s a lot of classism in the system, right? And people from working class communities or economically underprivileged communities are less likely to be able to get jobs in you know, mental health care, even though probably the majority of clients who really need it are also from economically stressed backgrounds.”

- Female, 58

#### Theme 2: Familiarity with task-sharing in other settings.

Many participants described finding task-shared mental health care to be acceptable because they reported having experienced it in other clinical settings previously.

“I guess in my brain, the way you said it kind of equates to me the equivalent of like, if I go to my doctor and my doctor uses a CNA [certified nursing assistant] or something, physician assistance a PA whatever, as their like go to if they’re not there. I very rarely have had a problem with that [task-sharing] in my past, so if it’s similar to that, then I’d probably be fully on board.”

- Male, 57

Several participants also expressed familiarity and comfort with the model based on seeing it in other non-clinical, community settings, including construction and teaching:

“Yeah, it all makes sense. I kind of think of it as I’d worked construction for 10 years, and the Union construction trade often has the apprentice journeyman, apprentice relationship. So, I kind of think of it like that”

- Other gender, 39

#### Theme 3: Questions related to task-sharing mental health care.

Despite their openness to task-shared mental health care, participants had questions about the feasibility of the model that will be important to address. Participants expressed questions and confusion about how they would be appropriately referred to more advanced providers if their needs were or became too complex for the task-shared provider to manage.

“With any quantity over quality situation, not to say that the [bachelor’s-level] therapists aren’t, you know, quality, of course, just that there are a lot more of them. There’s obviously the worry of well, what if I need something more intensive? And you know, obviously, then, I could go to get referred [to a specialist]. But at the same time there’s the worry that maybe these [bachelor-level] therapists just think that I’m unfixable and will just drop my case, you know.”

- Female, 26

“What if it was a emergency situation? You know what I mean? What if it was like a crisis?”

- Female, 49

Also related to safety, some participants wanted to better understand how supervision of task-shared mental health providers works. One participant explained how insufficient supervision was of particular concern because it could cause harm to patients:

“…But you know to really make sure that the education requirements and the training requirements and …whoever’s supervising [the bachelor-level provider] has a tight enough control of the situation that they don’t inadvertently do harm because they don’t have the skills.”

- Female, 70

Participants also had questions about being referred on to see other providers in scenarios outside of crisis situations, for example if they felt like the provider was not a good fit for them:

“A question I would have, or maybe a concern I would have is, if you know, for example, me and the provider that I was referred to—the task share provider you know—say we didn’t mesh, like sometimes you see a provider, and…you don’t connect and especially with therapy, I would want to know if I had other options. You know, whether that be seeing another task share provider, or, you know, being put on a wait list for like certified [specialist] provider. Kind of knowing what that process looks like would be helpful for me, I think.”

- Female, 27

In a few instances, participants expressed hesitation about the concept of a stepped care model in general. For example, some expressed concerns that a stepped care model could inadvertently lead to delaying their care. They were concerned that, if they ultimately needed to be referred from a task-shared provider to a more advanced provider, then they would have wasted time during which they could have been on the waitlist to see a mental health specialist in the first place:

“So like maybe [the patient] waited like a month to see [a task-shared provider]… and then, now that patient has to wait like another 6 months, which, if they would have just done it from the beginning, that would have already had a month off of the waiting list they could have done if they made the appointment. And then they’d get frustrated because they’re like, okay, well, like, I already waited a month for this appointment. And it’s not even like you can’t even do it now. I have to wait 6 months more. You might lose that patient.”

- Female, 46

Participants also wondered about the cost associated with seeing a task-shared mental health provider. Many said they expected the cost of seeing this provider would be less than a traditional mental health provider, which they viewed as positive:

“I assume that that the cost of seeing [a task-shared provider] is gonna be less than seeing you know somebody with a master’s or a PhD, so I, I think access is going to increase, which is great.”

- Male, 63

In some interviews, participants questioned whether task-shared providers would be younger compared to traditional mental health providers, and some older participants reported concerns about the acceptability of being seen by such young providers for their mental health.

“Age, so it might be hard for older patients to come in to speak with the younger person, because they might feel like you have no idea what life is like yet, or something”

- Female, 52

Lastly, when asked what would increase the acceptability of receiving care from a task-shared provider, several participants mentioned that being able to read a profile or learn about the providers background in advance or in the first session would be helpful.

“Basically, just like a short little, maybe a bio that like humanizes them a little bit like I’m [name]. I’m doing counseling because of this, and a little bit about this and this, and I don’t know something quirky like this is my hobby when I’m not doing work.

- Female, 28

### Quantitative findings

Most participants reported caring “a moderate amount” or “a lot” about the training backgrounds of mental health providers. Most participants also reported moderate to strong support for receiving care from a task-shared mental health provider. Please see Table 1 for complete survey results.

**Table 1:** Frequency and proportion on mhIST items.

	n (%)				
Item	Not at all	A little bit	A moderate amount	A lot	Don’t know
1. Interest in provider training background	1 (2.6)	3 (7.7)	11 (28.2)	24 (61.5)	0 (0)
2. Willingness to receive mental health treatment from a bachelor’s level provider	1 (2.6)	5 (12.8)	14 (35.9)	16 (41.0)	3 (7.7)
3. A bachelor’s level provider can address mental health needs	1 (2.6)	8 (20.5)	15 (38.5)	12 (30.8)	2 (5.1)
4. A bachelor’s level provider is qualified to deliver mental health care	2 (5.1)	8 (20.5)	17 (43.6)	12 (30.8)	0 (0)
5. My clinic is a good place for bachelor’s level providers to provide mental health care	1 (2.6)	5 (12.8)	14 (35.9)	15 (38.5)	4 (10.3)
6. Bachelor’s level providers are appropriate to help people with problems like me	4 (10.3)	7 (17.9)	11 (28.2)	17 (43.6)	0 (0)

### Integration of qualitative and quantitative data

Overall, when examined together, the quantitative and qualitative data converged, with participants reporting moderate to strong support for receiving care from a task-shared mental health provider across both data sources. This strengthens the finding provided by each data source alone. There was one key area of divergence between the qualitative and quantitative data. Quantitatively, participants reported moderate to strong interest in the training background of their mental health providers (i.e., type of degree, level of education), but in qualitative interviews shared that they didn’t understand mental health provider training backgrounds. This divergence suggests an area in which participant perspectives may not be clear.

## Discussion

These findings highlight the overall perceived acceptability and appropriateness of a task-shared mental health provider role to potential patients in urban, pre-implementation settings in WA, which is promising considering recent legislative reform that expands care delivery to bachelor’s level roles in the state and is being actively pursued and considered in other US states [27,28,30]. Participant responses to mhIST items demonstrated moderate to strong support for receiving care from a task-shared mental health provider. Qualitative interviews revealed that potential patient openness to this innovation was driven largely by their concern about mental health access issues and was bolstered by their existing familiarity with task-sharing from other clinical and community domains. However, findings also highlighted several areas in which questions about the feasibility of novel task-sharing and stepped care mental health care models may be necessary to address to strengthen buy-in among potential US patients less familiar with these models.

First, among many participant interviews, it was evident that there was limited clarity about mental health provider credentials and educational pathways in general. Quantitatively, almost 90% of participants reported “a moderate amount” or “a lot” to the question “how much do you care about the training background of your mental health provider?”, yet qualitatively, potential patients reported that they were not familiar with mental health provider training and education requirements. This limited level of understanding may have contributed to participant openness to seeing a new type of provider in this study, but such confusion could also lead to hesitation among other potential patients in the future. While a lack of understanding about mental health provider credentials is likely not unique to the United States, the US does have more types of mental health provider credentials than many other settings [55] and patients operate as consumers in the US healthcare system more than in many other healthcare systems (i.e., able to “shop” for providers [56]). Therefore, in a context with deployment of bachelor’s level mental health providers at scale, patient education about types of providers may be necessary to 1) help patients make informed decisions about their care and 2) ensure sufficient buy-in for bachelor’s level providers among other options.

Participants also brought up several key questions about stepped care models, which have traditionally been less used across the US health care system than in non-US systems [57]. Participants wanted to understand how supervision functioned within these models, whether they would be appropriately referred to more advanced providers if they were in crisis, their needs became too complex for the BHSS to manage, or they simply preferred to be seen by a different provider. In a few cases, participants were concerned that if they were ultimately referred to another mental health provider, they would have “wasted time” with the BHSS due to the inherent structure of a stepped care model. Careful health system planning will be critical to ensure care pathways and “step up” protocols are clearly defined and well-implemented as BHSS roles are introduced [58]. Patient education materials describing how these models function may also be important to develop. Due to the decentralization of the US healthcare system each health system will have to develop its own care pathways and patient education materials. However, implementation science researchers can help create core models for dissemination, building from the much more extensive literature on task-sharing mental health services in low- and middle-income countries [59–62].

Participants also made several assumptions about BHSS providers. Participants thought BHSS services would cost less than those of traditional mental health providers due to their more junior status, that they would be younger than their patients, and that they would be willing to disclose information about themselves. Several potential patients suggested that BHSSs could provide personal biographies about themselves and share their motivation for becoming a BHSS in advance of or at the first session to help increase patient confidence. While informed consent is always conducted at the start of treatment, brief provider biographies are sometimes provided within healthcare systems, and there are models of self-disclosure of lived experiences available from the peer support literature [63,64], the BHSS is not intended to be a peer support role and maintaining role clarity is important for the success of workforce capacity building projects within health and human services [65,66]. Further, the degree to which this information does actually increase patient confidence and the feasibility and acceptability within the health system could be important to understand further. Preliminary data collected by our team from the initial graduating classes of BHSSs in WA also indicates that many students begin the program from non-traditional educational backgrounds and may be the same age or older than traditional mental health providers. However, that may not be true at scale based on the mean age of college students in the US [67]. The implications of provider age on therapeutic alliance with patients are mixed in the empirical literature but this topic is worthy of future consideration, particularly among training programs [68].

Additionally, because new BHSS providers would have recently been in school, participants expected that BHSSs would be informed by more current mental health information. While this may initially be true, the availability of high-quality continuing education programs appropriately tailored to bachelor’s level providers will be necessary to ensure ongoing access to current mental health information. Several participants also thought that BHSSs would be less burnt out than traditional mental health providers, based on their belief that they would be younger and newer to their role. The international literature has identified that burnout is unfortunately as or more common in task-sharing mental health care models as in traditional mental health care models [69,70] and health systems need to plan carefully for professional development and wellness programs for task-shared mental health providers [69–71].

## Limitations

Findings should be interpreted considering study limitations. First, these interviews were conducted prior to the initial rollout of the BHSS in WA being completed. Therefore, participants were asked to consider a hypothetical rather than provide feedback on an actual experience. However, the sample was representative of patients likely to be seen by the BHSS, given that participants had anxiety and/or depression. Further, participants’ familiarity with mental health providers, in most instances multiple providers, enabled them to provide an informed perspective on their perceived comfort level with this new role. Additionally, the mixed-methods approach enabled a robust method of assessing convergence and strength of the data collected.

Although we used methods used previously by our team to gather input on an innovation prior to its introduction [72], perspectives on acceptability and feasibility may differ and be more enriched once patients actually receive task-shared care, and we therefore recommend future research of this role and its implementation after rollout. Notably, perspectives of these treatment-engaged participants may also not generalize to patients seeking mental health care for the first time. Furthermore, further research with patients with other conditions, aside from depression and anxiety, is an important future direction as the BHSS is not limited strictly to working with patients with these common diagnoses. Lastly, we collected data from participants able to speak English and seeking services in King County, the most urban part of the state. Understanding the perspectives of patients in other, more rural areas, where task-shared roles may be more likely to be deployed due to well-documented rural mental health provider staffing challenges [4,73], is also important.

### Implications for practice

A primary finding of our study is that the BHSS role is deemed appropriate and acceptable by potential patients, which implies a possible successful rollout of the new role. When preparing to integrate task-shared mental health roles, we recommend that health systems and policymakers be prepared to develop patient education on training pathways and roles and responsibilities, supervision structures, and stepped-care models to help facilitate patient buy-in. Additionally, continuing education and provider wellbeing programs must be developed to ensure these roles are sustainable.

## Conclusions

Participants expressed an overall willingness to receive psychological services from a bachelor’s level mental health provider, which is promising considering recent reform introducing these roles in WA and the general momentum behind task-sharing in mental health care. However, participant questions about these roles and the larger stepped-care model they are situated within reveal important areas to address when preparing to integrate these roles into healthcare settings and for educational programs to consider as they aim to train providers well prepared to engage patients.

## Supporting information

S1 File Consolidated criteria for reporting qualitative studies (COREQ): 32-item checklist. (DOCX)

S2 File Good Reporting of A Mixed Methods Study (GRAMMS) checklist. (DOCX)

S3 File Interview Guide. (DOCX)

S4 File Brief Survey. (DOCX)
