# Supplementary material for: Exploring patient perspectives on a new task-shared behavioral health role in Washington State
Source: PLOS Ment Health. 2026 Jun 29;3(6):e0000606. doi: 10.1371/journal.pmen.0000606 (PMC13313332; doi:10.1371/journal.pmen.0000606)
Supplement: S2 File — (DOCX) [file pmen.0000606.s002.docx]

**S2 File: Good Reporting of A Mixed Methods Study (GRAMMS) checklist**

| Guideline | Section and page |
| --- | --- |
| Describe the justification for using a mixed methods approach to the research question | DONE |
| Describe the design in terms of the purpose, priority and sequence of methods | 5-6 |
| Describe each method in terms of sampling, data collection and analysis | 5-6 |
| Describe where integration has occurred, how it has occurred and who has participated in it | 6 |
| Describe any limitation of one method associated with the presence of the other method | N/A |
| Describe any insights gained from mixing or integrating methods | 14-15 |
