# Supplementary material for: Exploring patient perspectives on a new task-shared behavioral health role in Washington State
Source: PLOS Ment Health. 2026 Jun 29;3(6):e0000606. doi: 10.1371/journal.pmen.0000606 (PMC13313332; doi:10.1371/journal.pmen.0000606)
Supplement: S3 File — (DOCX) [file pmen.0000606.s003.docx]

**S3 File: Interview Guide**

*Note: This document is meant to be a guide for the facilitator to structure the interviews; however, the facilitator is encouraged to follow the participants responses and probe to gather as accurate a sense of their perspectives as possible. The primary topic areas will stay the same, but the specific questions we expect to change based on information shared during the respective interviews.*

**Understanding patient perspectives on potential task-shared behavioral health roles in Washington State.**

1. Do you tend to understand the educational and training backgrounds of your mental health providers?
2. Do you tend to understand how your mental health providers work with your other healthcare providers?
3. Would you like to learn more about the educational and training backgrounds of your mental health providers?
4. Would you like to learn more about how your mental health providers work with your other healthcare providers?

Today I would like to get your perspectives on something called task-shared mental health. This means receiving care from a provider with less training in mental health, often under supervision from providers with more experience. Task-shared providers see stable patients, rather than patients with complex needs or who are in crisis. The intention of task-sharing is to help reduce wait times and increase access to care for patients. For the right type of patient this has been shown to be as effective as care by a traditional mental health provider. It has primarily been developed in international health systems with more severe staffing shortages and is now being tested in the United States.

1. Based on what I have just shared, would you be willing to see a task-shared provider?
   1. Why or why not?
   2. What questions, if any, do you have about this type of provider?
      1. Note: You are not going to actually answer these questions now, we just want to collect them. If the participant seems to really want answers to their questions tell them you are able to share a case example that you hope will clarify things and if not you can try to answer their questions after.
   3. Do you think a task-shared provider would be able to help you with your mental health?
      1. Why or why not?

Now I will describe what this could look like in Washington state. A patient, Kim, shares with her primary care provider at her annual visit that she has been feeling down most of the time and has been struggling to sleep. After conducting a brief screener, her primary care provider identifies that Kim has mild depression and that she would benefit from a course of therapy. After discussing this with Kim and learning that she is open to trying therapy to help with these symptoms, the primary care provider refers Kim to Charles, a colleague at the clinic. Charles has a 4-year college degree in providing mental health care and has obtained a credential with the Department of Health to provide mental health care under supervision from another provider. They do not have a master’s (2 years study post college) or a doctoral degree (6 years study post college), which a traditional mental health care provider would. However, they can meet with Kim much sooner than a provider with a master’s or doctoral degree. And if Kim ends up needing more help than Charles is able to provide, Charles would then refer Kim to those more traditional mental health services.

1. Would you be willing to see a task-shared provider for your mental health after hearing this example?
   1. Why or why not?
   2. What questions, if any, do you have about this type of provider?
   3. Do you think they would be able to help you with your mental health?
2. What are the key advantages, if any, that you see about seeing a provider like Charles?
3. What are the key disadvantages, if any, that you see about seeing a provider like Charles?
4. Do you think most patients like you would be comfortable seeing a provider like Charles?
5. What other factors do you consider when choosing a mental health provider?
   1. Are there any other factors you consider?

Thank you for completing the interview, this was very helpful. Is there anything else we haven’t talked about that you would like to add?

**Remind them they will now electronically receive the gift card and the quantitative survey to complete (for another gift card).**
