# Supplementary material for: Exploring patient perspectives on a new task-shared behavioral health role in Washington State
Source: PLOS Ment Health. 2026 Jun 29;3(6):e0000606. doi: 10.1371/journal.pmen.0000606 (PMC13313332; doi:10.1371/journal.pmen.0000606)
Supplement: S4 File — (DOCX) [file pmen.0000606.s004.docx]

**S4 File: Brief Survey**

1. How much do you care about the training background of your mental health providers?

2. Overall, how willing would you be to receive mental health treatment from a bachelor’s level provider?

3. Do you think a bachelor’s level provider would have the abilities to address your mental health needs?

4. Do you think a bachelor’s level provider would be qualified to deliver your care?

5. Is the clinic where you receive care a good place for bachelor’s level providers to be providing mental health care?

6. Do you think bachelor’s level providers are appropriate to be helping people with problems like yours?
