# Supplementary material for: Exploring patient perspectives on a new task-shared behavioral health role in Washington State
Source: PLOS Ment Health. 2026 Jun 29;3(6):e0000606. doi: 10.1371/journal.pmen.0000606 (PMC13313332; doi:10.1371/journal.pmen.0000606)
Supplement: Attachment — Submitted filename: Revision_letter_final.docx [file pmen.0000606.s006.docx]

April 14^th^, 2026

Lambert Zixin Li, PhD

Academic Editor

PLOS Mental Health

Dear Dr. Li:

Thank you for the positive and constructive feedback regarding our manuscript titled “Exploring patient perspectives on a new task-shared behavioral health role in Washington State.” We attach a revised version with changes in track changes as well as a new, clean version of the manuscript. Below we list all comments we received in the review and detail our revisions in response.

**Journal Requirements:**

1. Please describe in your methods section how capacity to provide consent was determined for the participants in this study. Please also state whether your ethics committee or IRB approved this consent procedure. If you did not assess capacity to consent please briefly outline why this was not necessary in this case.

**RESPONSE:** Thank you for this question. We have now added this on page 5: “As approved by the UW Human Subjects Division, patients with documentation of cognitive impairment in the medical record were excluded from extraction.”

2. Please amend your detailed Financial Disclosure statement. This is published with the article. It must therefore be completed in full sentences and contain the exact wording you wish to be published.
i. Please clarify all sources of financial support for your study. List the grants, grant numbers, and organizations that funded your study, including funding received from your institution. Please note that suppliers of material support, including research materials, should be recognized in the Acknowledgements section rather than in the Financial Disclosure.
   ii. State the initials, alongside each funding source, of each author to receive each grant. For example: "This work was supported by the National Institutes of Health (####### to AM; ###### to CJ) and the National Science Foundation (###### to AM)."
   iii. State what role the funders took in the study. If the funders had no role in your study, please state: “The funders had no role in study design, data collection and analysis, decision to publish, or preparation of the manuscript.”
   iv. If any authors received a salary from any of your funders, please state which authors and which funders.

**RESPONSE:** We have amended our statement and provided additional detail as requested.

3. Please ensure that your Ethics Statement is available in its entirety at the beginning of your Methods section, under a subheading 'Ethics Statement'. It must include:

i) The name(s) of the Institutional Review Board(s) or Ethics Committee(s)

ii) The approval number(s), or a statement that approval was granted by the named board(s)

iii) (for human participants or donors) - A statement that formal consent was obtained (must state whether verbal/written) OR the reason consent was not obtained (e.g., anonymity).

**RESPONSE:** We have now reformatted the beginning of Methods section to have an Ethics Statement subheading. This includes the name of the IRB (University of Washington Human Subjects Division), the approval number, and description of the format of consent.

4. We have noticed that you have uploaded Supporting Information files, but you have not included a list of legends. Please add a full list of legends for your Supporting Information files after the references list.

**RESPONSE:** Apologies. We now include a list of legends after the reference list.

5. In the online submission form, you indicated that “The data that support the findings of this study are available from the corresponding author, ALR, upon reasonable request. We did not obtain consent from participants to deposit data, which is largely comprised of qualitative interviews, in a public repository.”.

All PLOS journals now require all data underlying the findings described in their manuscript to be freely available to other researchers, either 
1. In a public repository, 
2. Within the manuscript itself, or 
3. Uploaded as supplementary information.

This policy applies to all data except where public deposition would breach compliance with the protocol approved by your research ethics board. If your data cannot be made publicly available for ethical or legal reasons (e.g., public availability would compromise patient privacy), please explain your reasons by return email and your exemption request will be escalated to the editor for approval. Your exemption request will be handled independently and will not hold up the peer review process, but will need to be resolved should your manuscript be accepted for publication. One of the Editorial team will then be in touch if there are any issues.

**RESPONSE:** We have responded to the editor by email as requested.

6. If the reviewer comments include a recommendation to cite specific previously published works, please review and evaluate these publications to determine whether they are relevant and should be cited. There is no requirement to cite these works unless the editor has indicated otherwise.

**RESPONSE:** We have now included relevant additional publications, including those on topics suggested by the reviewers.

**Editor Comments:**

1. Dear Authors, Thank you for submitting your manuscript. The topic addressed in this study is important and potentially relevant to the journal’s readership. After careful consideration, however, the reviewers have raised several substantive concerns regarding the conceptual framing, methodological transparency, and interpretation of the findings.
   For these reasons, the manuscript would benefit from significant revision before it can be reconsidered. I encourage the authors to carefully review the attached comments and revise the manuscript accordingly. I hope you find the feedback constructive and helpful in strengthening the work.

**RESPONSE:** We are very pleased you have invited this manuscript for revision and resubmission and are grateful for the very helpful comments provided by the reviewers. These comments have helped us to substantially improve the manuscript.

**Reviewer 1:**

1. This manuscript presents an important study examining patient acceptability of bachelor’s-level, task-shared mental health providers within a stepped-care model in Washington State. The topic is timely and policy-relevant; however, several conceptual and methodological aspects require strengthening to meet the rigor increasingly expected in contemporary mental health services research.

**RESPONSE:** Thank you for your comments, particularly around conceptual and methodological aspects. These have helped us to strengthen the paper, and we address them in turn below.

2. Recommendations: The manuscript presents robust evidence that patients are generally open to receiving care from bachelor’s-level, task-shared mental health providers. To strengthen its contribution, the authors should more clearly articulate what this study adds beyond confirming acceptability, such as adding in the discussion section about nuanced patient concerns around supervision, escalation, and role clarity that may inform implementation design within stepped-care models in the US health systems.

**RESPONSE:** Thank you for this comment. Though this topic is generally novel in the United States, we agree that this paper adds to a larger literature. We have now expanded details throughout the discussion about considerations specific to the US system to highlight the unique contributions of this paper. For example, on page 18 we write: “While a lack of understanding about mental health provider credentials is likely not unique to the United States, the US does have more types of mental health provider credentials than many other settings (55) and patients operate as consumers in the US healthcare system more than in many other healthcare systems (i.e., able to “shop” for providers (56)). Therefore, in a context with deployment of bachelor’s level mental health providers at scale, patient education about types of providers may be necessary to 1) help patients make informed decisions about their care and 2) ensure sufficient buy-in for bachelor’s level providers among other options.”

3. While the manuscript is well grounded in the US policy and systems literature, its discussion would benefit from inclusion of evidence from low- and middle-income countries, where task-sharing has been extensively implemented and evaluated at scale. Drawing selective parallels could contextualize patient concerns observed in this study and highlight transferable lessons for training, supervision, referral pathways, and patient education.

**RESPONSE:** Thank you for this point. We agree that the US has much to learn from the international literature on this topic and have now added in additional references and discussion of this literature in the discussion. For example, on page 19 we write: “However, implementation science researchers can help create core models for dissemination, building from the much more extensive literature on task-sharing mental health services in low- and middle-income countries (59–62).” And, on page 20, we write: “The international literature has identified that burnout is unfortunately as or more common in task-sharing mental health care models as in traditional mental health care models (69,70) and health systems need to plan carefully for professional development and wellness programs for task-shared mental health providers (69–71).”

4. As participants provided feedback prior to full BHSS rollout, acceptability should be consistently framed as perceived or anticipatory. Minor language refinements would help avoid over-interpretation and ensure alignment between the study design and its conclusions. Briefly noting how perspectives may change once patients actually receive task-shared care would also help guide interpretation and future research.

**RESPONSE:** Thank you for noting this. We agree minor language refinements were necessary here and have made these adjustments throughout. We also now discuss how perspectives may change through direct experience on page 21: “Although we used methods used previously by our team to gather input on an innovation prior to its introduction (72), perspectives on acceptability and feasibility may differ and be more enriched once patients actually receive task-shared care, and we therefore recommend future research of this role and its implementation after rollout.”

5. The discussion identifies several actionable issues (e.g., patient education on provider roles, supervision structures, and stepped-care pathways). Consolidating these insights into a short, structured set of implementation considerations would enhance the manuscript’s practical value for health systems and policymakers preparing to integrate task-shared mental health roles.

**RESPONSE:** Thank you for this suggestion. We have worked to consolidate these insights to increase their practical use. They now appear on page 21 in a section titled “Implications for practice.”

6. While the manuscript presents rich qualitative data, its contribution could be further strengthened by explicitly linking key themes (e.g., uncertainty around credentials, supervision, and stepped-care pathways) to established implementation constructs such as acceptability, appropriateness, and feasibility, and by clarifying the transferability of these anticipatory findings. Specifically, articulating the contexts in which these results are most applicable (e.g., urban, pre-implementation settings) and how patient perspectives may evolve following real-world exposure to task-shared care would support more theory-informed interpretation and better guide future research.

**RESPONSE:** Thank you for this feedback. Theme 1 was phrased in alignment with acceptability previously, but we now more accurately present it in alignment with appropriateness and acceptability and use this language more consistently throughout all themes. Theme 3 is now tied primarily to feasibility. We also make explicit the specific contexts in which these findings currently apply at the beginning of the discussion, in lines 346-357. In response to point 4 above we also now discuss how perspectives may evolve through real-world exposure.

**Reviewer 2:**

1. Thank you for the opportunity to review this manuscript. The study addresses an important topic and contributes to the existing literature by exploring patient perspectives on a new task-shared behavioral health role. The manuscript is generally well organized, and the topic is timely and significant. The authors provide a clear background and identify a meaningful research gap that the study aims to address.

**RESPONSE:** Thank you for these comments on the significance and overall strength of this manuscript.

2. However, there are several areas that require clarification and strengthening. First, the study is described as a mixed-methods design, yet the abstract’s results section does not report any quantitative findings or clearly reference the quantitative component of the study. The authors should present the relevant findings if the study indeed includes a quantitative component.

**RESPONSE:** Thank you for noting this. We now report on the quantitative measure and more clearly discuss the study as mixed methods in the abstract.

3. Second, the study participants appear to have been primarily diagnosed with depression and anxiety. A clearer explanation is needed regarding why these diagnostic groups were selected. It would be helpful for the authors to clarify whether the BHSS will specifically work with only the individuals with these conditions, or whether the exclusion of other populations—such as individuals with substance use disorders or schizophrenia—was intentional.

**RESPONSE:** Thank you for this question. The BHSS will not only work with patients with depression and anxiety but due to the prevalence rates of these conditions in the population and relative brevity of BHSS training they are expected to be common presenting problems assigned to this cadre. We have clarified this on page 5 (please see next point for quote). We have also added further research with patients with other conditions as an important future direction in our discussion.

4. Additionally, a more detailed description of the Behavioral Health Support Specialist (BHSS) role in Washington State would enhance the reader’s understanding of the context and scope of this position. Providing information about the responsibilities and integration of this role within the healthcare system would help readers better interpret the study findings.

**RESPONSE:** Thank you for this point. We have added additional detail on the role and responsibilities of the BHSS on page 5 in response to this point and point 6 from Reviewer 3. It now reads: “The WA Department of Health (DOH) specified that the BHSS may work under supervision delivering evidence-based psychological interventions (e.g., behavioral activation, motivational interviewing) in any setting offering mental health and behavioral health services in WA to maximize the broad utility of the role (31). Due to the prevalence of common mental disorders and the relative brevity of BHSS training they are expected to work primarily with patients with mild to moderate depression and anxiety in outpatient settings offering behavioral health services (e.g., primary care mental health, community mental health agencies) (32).”

5. The manuscript would also benefit from additional clarification regarding the study setting. Specifically, the authors should indicate the types of clinics or healthcare settings from which participants were recruited.

**RESPONSE:** Thank you for this point. We have added additional detail on the recruitment settings on pages 6-7. It now reads: “Due to the emerging nature of the BHSS at that time (i.e. no BHSSs had yet graduated from their training programs), we sampled potential patients that, based on their clinical characteristics, may be likely to receive care from a BHSS in the future at clinics that may be likely to employ a BHSS. In alignment with the Washington State Department of Health and developers of the BHSS role, we defined clinics that may be likely to employ a BHSS in the future as those providing mental health care integrated within primary care settings and managing lengthy waitlists for mental health services.”

6. Finally, it would be helpful for the authors to specify the language(s) in which the interviews were conducted. Given the diversity of the United States population, clarifying whether interviews were conducted only in English or in multiple languages is important. If participants who spoke other languages were excluded, this should be clearly stated and discussed as a potential limitation.

**RESPONSE:** Thank you for this important comment. We did only conduct interviews in English due to limitations of our interviewers. We have now acknowledged this as a limitation on page 21.

7. Overall, the manuscript would benefit from revisions to enhance clarity, methodological rigor, and interpretation of the findings. I encourage the authors to address the comments provided to strengthen the manuscript.

**RESPONSE:** Thank you for these helpful comments to enhance clarity, methodological rigor, and support interpretation. They have helped strengthen this manuscript considerably.

**Reviewer 3:**

1. Thank you for the opportunity to contribute to the review of this manuscript. This study addresses the critical issue of task sharing among (mental) health professionals at a time when many countries are concerned about the shortage of human resources in this field. First, I want to congratulate the authors on the quality of their work. Below, I have made some suggestions for improvement, which I leave to their discretion.

**RESPONSE:** Thank you for your positive comments about this manuscript and its importance. We appreciate your suggestions and have incorporated them throughout, which we describe below.

2. Abstract The manuscript sometimes refers to “potential patients” and sometimes to “patients.” It is important to harmonize this concept (throughout the manuscript) to comply with what is indicated in the title.

**RESPONSE:** Thank you for noting this. We have harmonized language throughout.

3. This is intended to be a mixed-methods study, but the abstract does not contain any quantitative results. Please add quantitative findings if possible.

**RESPONSE:** Thank you, Reviewer 2 also noted this (point 2), and we have addressed this.

4. What does BHSS mean? Please avoid acronyms in the abstract.

**RESPONSE:** The BHSS refers to the Behavioral Health Support Specialist, which we had named separately but not clearly related to the acronym as we should have. We have now corrected this and reduced use of acronyms in the abstract.

5. Introduction

It is stated that task-sharing has been chosen as a strategy to address the shortage of advanced mental health professionals, but there are no statistics to support this claim. Please provide some if they exist.

**RESPONSE:** Thank you for this feedback. We have now added additional references and statistics to support the rationale for task-sharing on page 4. We write “In the United States, federally designated Mental Health Care Health Professional Shortage Areas (HPSAs), defined by the ratio of mental health professionals relative to the population, were present in every US state (8)” and “using task-sharing to introduce more providers into the mental healthcare system is anticipated to expand access to mental healthcare by introducing more staff to the system and allowing the more limited group of advanced mental health professionals to focus their expertise on more complex cases (23).”

6. The manuscript states that the Washington State Department of Health has clarified the BHSS framework and that formative work has been done to define their role. However, it is important to clearly present in this manuscript the tasks shared with BHSS as clinicians. In addition, it is necessary to indicate whether these tasks would be performed in primary care settings or specialized hospitals.

**RESPONSE:** Thank you. Reviewer 2 also asked for additional information on the roles and responsibilities that the BHSS can work in (point 4) and the most salient settings (point 5). We have added additional information on pages 5-7.

7. Materials and Methods
   It is stated that the study was presented in accordance with COREQ and GRAMMS guidelines, which is excellent. However, the manuscript, which refers potential readers to supplementary files, does not clearly and separately present the various traditional subsections, such as study design, study setting, and data collection procedure. I strongly advise providing the essential information for each of these subsections before referring to the supplementary files.

**RESPONSE:** Thank you for this feedback. We have increased the number and specificity of our section headings throughout, which we hope is clearer.

8. The “Participants” subsection provides information on both the participants and the sampling procedure. It could be renamed “Participants and Sampling.”

**RESPONSE:** Thank you for this feedback. We have renamed this.

9. Some information is mixed together in a single subsection, such as the “Participants” subsection, which includes both participants, the data collection period, etc. For clarity, I suggest clearly separating this information into corresponding subsections.

**RESPONSE:** Thank you for this feedback. We have increased the number and specificity of our section headings throughout.

10. I suggest providing operational definitions of the concepts of “acceptability” and “appropriateness” in the manuscript and then citing the reference, such as Proctor et al. (44), if these are the definitions adopted, instead of simply referring to the reference.

**RESPONSE:** We agree and now include definitions along with the reference on page 7-8.

11. The manuscript describes the descriptive statistical analyses that were performed. However, it provides little or no clear information about the qualitative analyses performed. I suggest clarifying this information.

**RESPONSE:** We wish to highlight our description of the qualitative analysis and integration of qualitative and quantitative data on pages 8-9. Increased specificity in our subheadings will clarify this for readers as well.

12. If the possible responses to the quantitative data collection tool ranged from 1 = Not at all to 4 = Very much and included “I don't know,” this implies that the variables were qualitative (i.e., categorical variables). If this is the case, it is generally accepted that, for this type of variable, frequencies and proportions are estimated and then expressed as percentages. Because the mean and standard deviation were calculated, the authors would do well to explain in the manuscript how they transformed the qualitative variables into quantitative variables to estimate these measures of central tendency and dispersion.

**RESPONSE:** Thank you for this point. We now present these data as frequencies and percentages in the text and Table 1.

13. Results

For the description of the participant profile, as the sample size (N = 40) is already mentioned, I suggest simply indicating the percentages when presenting the results and deleting the numbers (n = x), as this gives an impression of repetition.
These “Thirty-eight participants completed the interview with a usable audio-recording. Thirty-nine participants completed the survey.” statements do not constitute participant characteristics. They can be moved to the Methods section.

**RESPONSE:** Thank you for these comments. We now present only percentages for our demographic description of the sample. We also have removed the information on completion of the survey and the interview and placed this information in the Methods section.

14. Please clarify the quantitative findings. Stating “... aligning with moderate to strong interest in the training backgrounds of their mental health providers and moderate to strong support for receiving care from a task-shared mental health provider.” is not very clear to readers.

**RESPONSE:** Thank you for this feedback. We have rephrased this sentence to realign with the way we now present the quantitative data and to clarify our meaning. On page 16 it now reads: Most participants reported caring “a moderate amount” or “a lot” about the training backgrounds of mental health providers. Most participants also reported moderate to strong support for receiving care from a task-shared mental health provider. Please see Table 1 for complete survey results.

15. In Table 1, is it possible to replace the questions asked participants with research variables? This would make these results a little easier to understand.

**RESPONSE:** Thank you for this suggestion. We have relabeled the rows in this table to make the items simpler and clearer. We now include the full survey in the supporting files.

16. The text in the “Integration of qualitative and quantitative data” subsection reads like a conclusion. Please present this subsection more clearly so that it reflects the mutual influence of the qualitative and quantitative findings.

**RESPONSE:** Thank you for commenting on the lack of sufficient clarity here. We have rewritten this section to increase its clarity and think it is much improved now. On page 17 it now reads: “Overall, when examined together, the quantitative and qualitative data converged, with participants reporting moderate to strong support for receiving care from a task-shared mental health provider across both data sources. This strengthens the finding provided by each data source alone. There was one key area of divergence between the qualitative and quantitative data. Quantitatively, participants reported moderate to strong interest in the training background of their mental health providers (i.e. type of degree, level of education), but in qualitative interviews shared that they didn’t understand mental health provider training backgrounds. This divergence suggests an area in which participant perspectives may not be clear.”

17. Discussion

I suggest elaborating a little more clearly on the strengths, limitations, and study implications and, if possible, making it a separate subsection.

**RESPONSE:** Thank you for this point. We now present limitations and study implications in separate subsections and more explicitly highlight strengths alongside limitations.

Thank you again for the feedback and for the opportunity to revise and resubmit. This feedback has helped us clarify key issues. Thank you as well for considering this manuscript further.

Authors
