# Supplementary material for: Exploring patient perspectives on a new task-shared behavioral health role in Washington State
Source: PLOS Ment Health. 2026 Jun 29;3(6):e0000606. doi: 10.1371/journal.pmen.0000606 (PMC13313332; doi:10.1371/journal.pmen.0000606)
Supplement: Attachment — Submitted filename: Revision_letter_5.18.docx [file pmen.0000606.s007.docx]

May 18^th^, 2026

Lambert Zixin Li, PhD

Academic Editor

PLOS Mental Health

Dear Dr. Li:

Thank you for the further comments regarding our manuscript titled “Exploring patient perspectives on a new task-shared behavioral health role in Washington State.” We attach a revised version with changes in track changes as well as a new, clean version of the manuscript. Below we list all additional comments we received in the review and detail our revisions in response.

**Editor:**

1. Thank you for submitting your revised manuscript. The paper has improved substantially and is now close to being suitable for publication. After considering the latest review and my own assessment, I would like to invite a minor revision to address a few remaining comments raised by one reviewer. Please revise the manuscript accordingly and provide a brief point by point response. I look forward to receiving the revised version.

**RESPONSE:** Thank you for this comment. We have appreciated the reviewer comments and agree the paper is much improved. We respond point by point to the remaining reviewer concerns below.

**Reviewer #2:**

1. Thank you for your thoughtful revisions to the initial version of the manuscript. The updated draft demonstrates meaningful improvement in clarity and organization. I appreciate the effort invested in addressing the earlier feedback.

**RESPONSE:** Thank you for this feedback. We appreciate your prior feedback and how it	your comments have helped us improve this manuscript.

2. I noted, however, that the manuscript indicates 40 participants were recruited, whereas the results section reports findings from 38 participants. Please clarify the disposition of the two participants who were not included in the final analysis, including whether they withdrew, were excluded, or had incomplete data. If applicable, providing possible reasons for withdrawal or exclusion would strengthen the methodological transparency of the study.

**RESPONSE:** Thank you for this question. We did have incomplete data from three participants – two in the interview and one in the survey. We have now made this clearer on page 8. For the interviews, in one case it was because the audio file was unusable due to background noise/technological issues. In the other case it was because the person did not finish the interview due to feeling ill. For the survey, only 39 of the 40 participants returned the survey despite sending several reminders.

3. In addition, although the interview guide has been included as an appendix, it would enhance the Methods section to incorporate a concise summary of the data collection tools and interview procedures. Briefly describing the structure of the interview guide and the major domains explored would improve readability and provide readers with a clearer understanding of the methodological approach without relying solely on the appendix.

**RESPONSE:** Thank you for this suggestion. We have added additional detail into our description of the methods on pages 7 and 8. It now reads: “Interview guides first asked participants about their understanding of and interest in mental health provider training backgrounds and cooperation with other providers. Next, since participants were unlikely to have had prior contact or familiarity with task-shared mental health providers, interview guides presented two vignettes of task-shared mental health care to participants before asking for feedback on acceptability and appropriateness of these roles. In alignment with the Proctor et al., definitions, acceptability was defined as the participants’ perception that the BHSS role was agreeable, palatable, or satisfactory, and appropriateness was defined as the participants’ perceived fit, relevance, or compatibility of this new role for their given clinic setting and their mental health needs (48). Lastly, interviews asked about perceived advantages and disadvantages of receiving care from this type of provider.”

**Reviewer #3**

1. Thank you for addressing all my concerns. I have nothing further to add. I hope this has helped to improve the quality of the manuscript.

**RESPONSE:** Thank you for your helpful feedback. Your comments have helped improve this paper and we are glad we have addressed all your concerns.

Thank you again for the feedback and for the opportunity to revise and resubmit.

Authors
