# Supplementary material for: Exploring patient perspectives on a new task-shared behavioral health role in Washington State
Source: PLOS Ment Health. 2026 Jun 29;3(6):e0000606. doi: 10.1371/journal.pmen.0000606 (PMC13313332; doi:10.1371/journal.pmen.0000606)
Supplement: Peer review history

# Peer review: Decision Letter 0

**DOI:** 10.1371/journal.pmen.0000606.r001

**Authors:** Lambert Zixin Li

9 Mar 2026

PMEN-D-26-00055

Exploring patient perspectives on a new task-shared behavioral health role in Washington State

PLOS Mental Health

Dear Dr. Rose,

Thank you for submitting your manuscript to PLOS Mental Health. After careful consideration, we feel that it has merit but does not fully meet PLOS Mental Health’s publication criteria as it currently stands. Therefore, we invite you to submit a revised version of the manuscript that addresses the points raised during the review process.

Please submit your revised manuscript by Apr 23 2026 11:59PM. If you will need more time than this to complete your revisions, please reply to this message or contact the journal office at mentalhealth@plos.org. When you're ready to submit your revision, log on to https://www.editorialmanager.com/pmen/ and select the 'Submissions Needing Revision' folder to locate your manuscript file.

Please include the following items when submitting your revised manuscript:

- A letter that responds to each point raised by the editor and reviewer(s). You should upload this letter as a separate file labeled 'Response to Reviewers'.
- A marked-up copy of your manuscript that highlights changes made to the original version. You should upload this as a separate file labeled 'Revised Manuscript with Track Changes'.
- An unmarked version of your revised paper without tracked changes. You should upload this as a separate file labeled 'Manuscript'.

Guidelines for resubmitting your figure files are available below the reviewer comments at the end of this letter.

We look forward to receiving your revised manuscript.

Kind regards,

Lambert Zixin Li, Ph.D.

Academic Editor

PLOS Mental Health

**Journal Requirements:**

1. Please describe in your methods section how capacity to provide consent was determined for the participants in this study. Please also state whether your ethics committee or IRB approved this consent procedure. If you did not assess capacity to consent please briefly outline why this was not necessary in this case.

2. Please amend your detailed Financial Disclosure statement. This is published with the article. It must therefore be completed in full sentences and contain the exact wording you wish to be published.

i. Please clarify all sources of financial support for your study. List the grants, grant numbers, and organizations that funded your study, including funding received from your institution. Please note that suppliers of material support, including research materials, should be recognized in the Acknowledgements section rather than in the Financial Disclosure.

ii. State the initials, alongside each funding source, of each author to receive each grant. For example: "This work was supported by the National Institutes of Health (####### to AM; ###### to CJ) and the National Science Foundation (###### to AM)."

iii. State what role the funders took in the study. If the funders had no role in your study, please state: “The funders had no role in study design, data collection and analysis, decision to publish, or preparation of the manuscript.”

iv. If any authors received a salary from any of your funders, please state which authors and which funders.

3. Please ensure that your Ethics Statement is available in its entirety at the beginning of your Methods section, under a subheading 'Ethics Statement'. It must include:

i) The name(s) of the Institutional Review Board(s) or Ethics Committee(s)

ii) The approval number(s), or a statement that approval was granted by the named board(s)

iii) (for human participants or donors) - A statement that formal consent was obtained (must state whether verbal/written) OR the reason consent was not obtained (e.g., anonymity).

4. We have noticed that you have uploaded Supporting Information files, but you have not included a list of legends. Please add a full list of legends for your Supporting Information files after the references list.

5. In the online submission form, you indicated that “The data that support the findings of this study are available from the corresponding author, ALR, upon reasonable request. We did not obtain consent from participants to deposit data, which is largely comprised of qualitative interviews, in a public repository.”.

All PLOS journals now require all data underlying the findings described in their manuscript to be freely available to other researchers, either

1. In a public repository,

2. Within the manuscript itself, or

3. Uploaded as supplementary information.

This policy applies to all data except where public deposition would breach compliance with the protocol approved by your research ethics board. If your data cannot be made publicly available for ethical or legal reasons (e.g., public availability would compromise patient privacy), please explain your reasons by return email and your exemption request will be escalated to the editor for approval. Your exemption request will be handled independently and will not hold up the peer review process, but will need to be resolved should your manuscript be accepted for publication. One of the Editorial team will then be in touch if there are any issues.

If the reviewer comments include a recommendation to cite specific previously published works, please review and evaluate these publications to determine whether they are relevant and should be cited. There is no requirement to cite these works unless the editor has indicated otherwise.

**Additional Editor Comments (if provided):**

Dear Authors,

Thank you for submitting your manuscript. The topic addressed in this study is important and potentially relevant to the journal’s readership. After careful consideration, however, the reviewers have raised several substantive concerns regarding the conceptual framing, methodological transparency, and interpretation of the findings.

For these reasons, the manuscript would benefit from significant revision before it can be reconsidered. I encourage the authors to carefully review the attached comments and revise the manuscript accordingly.

I hope you find the feedback constructive and helpful in strengthening the work.

Best regards,

Lambert Zixin Li, PhD

[Note: HTML markup is below. Please do not edit.]

Reviewers' comments:

Reviewer's Responses to Questions

**Comments to the Author**

1. Does this manuscript meet PLOS Mental Health’s publication criteria? Is the manuscript technically sound, and do the data support the conclusions? The manuscript must describe methodologically and ethically rigorous research with conclusions that are appropriately drawn based on the data presented.

Reviewer #1: Yes

Reviewer #2: Yes

Reviewer #3: Partly

**********

2. Has the statistical analysis been performed appropriately and rigorously?

Reviewer #1: Yes

Reviewer #2: N/A

Reviewer #3: No

**********

3. Have the authors made all data underlying the findings in their manuscript fully available (please refer to the Data Availability Statement at the start of the manuscript PDF file)?

The PLOS Data policy requires authors to make all data underlying the findings described in their manuscript fully available without restriction, with rare exception. The data should be provided as part of the manuscript or its supporting information, or deposited to a public repository. For example, in addition to summary statistics, the data points behind means, medians and variance measures should be available. If there are restrictions on publicly sharing data—e.g. participant privacy or use of data from a third party—those must be specified.

Reviewer #1: Yes

Reviewer #2: No

Reviewer #3: Yes

**********

4. Is the manuscript presented in an intelligible fashion and written in standard English?

PLOS Mental Health does not copyedit accepted manuscripts, so the language in submitted articles must be clear, correct, and unambiguous. Any typographical or grammatical errors should be corrected at revision, so please note any specific errors here.

Reviewer #1: Yes

Reviewer #2: Yes

Reviewer #3: Yes

**********

5. Review Comments to the Author

Please use the space provided to explain your answers to the questions above. You may also include additional comments for the author, including concerns about dual publication, research ethics, or publication ethics. (Please upload your review as an attachment if it exceeds 20,000 characters)

**Reviewer #1:**  This manuscript presents an important study examining patient acceptability of bachelor’s-level, task-shared mental health providers within a stepped-care model in Washington State. The topic is timely and policy-relevant; however, several conceptual and methodological aspects require strengthening to meet the rigor increasingly expected in contemporary mental health services research.

Recommendations:

•The manuscript presents robust evidence that patients are generally open to receiving care from bachelor’s-level, task-shared mental health providers. To strengthen its contribution, the authors should more clearly articulate what this study adds beyond confirming acceptability, such as adding in the discussion section about nuanced patient concerns around supervision, escalation, and role clarity that may inform implementation design within stepped-care models in the US health systems.

•While the manuscript is well grounded in the US policy and systems literature, its discussion would benefit from inclusion of evidence from low- and middle-income countries, where task-sharing has been extensively implemented and evaluated at scale. Drawing selective parallels could contextualize patient concerns observed in this study and highlight transferable lessons for training, supervision, referral pathways, and patient education.

•As participants provided feedback prior to full BHSS rollout, acceptability should be consistently framed as perceived or anticipatory. Minor language refinements would help avoid over-interpretation and ensure alignment between the study design and its conclusions. Briefly noting how perspectives may change once patients actually receive task-shared care would also help guide interpretation and future research.

•The discussion identifies several actionable issues (e.g., patient education on provider roles, supervision structures, and stepped-care pathways). Consolidating these insights into a short, structured set of implementation considerations would enhance the manuscript’s practical value for health systems and policymakers preparing to integrate task-shared mental health roles.

•While the manuscript presents rich qualitative data, its contribution could be further strengthened by explicitly linking key themes (e.g., uncertainty around credentials, supervision, and stepped-care pathways) to established implementation constructs such as acceptability, appropriateness, and feasibility, and by clarifying the transferability of these anticipatory findings. Specifically, articulating the contexts in which these results are most applicable (e.g., urban, pre-implementation settings) and how patient perspectives may evolve following real-world exposure to task-shared care would support more theory-informed interpretation and better guide future research.

**Reviewer #2:**  Thank you for the opportunity to review this manuscript. The study addresses an important topic and contributes to the existing literature by exploring patient perspectives on a new task-shared behavioral health role. The manuscript is generally well organized, and the topic is timely and significant. The authors provide a clear background and identify a meaningful research gap that the study aims to address.

However, there are several areas that require clarification and strengthening. First, the study is described as a mixed-methods design, yet the abstract’s results section does not report any quantitative findings or clearly reference the quantitative component of the study. The authors should present the relevant findings if the study indeed includes a quantitative component.

Second, the study participants appear to have been primarily diagnosed with depression and anxiety. A clearer explanation is needed regarding why these diagnostic groups were selected. It would be helpful for the authors to clarify whether the BHSS will specifically work with only the individuals with these conditions, or whether the exclusion of other populations—such as individuals with substance use disorders or schizophrenia—was intentional.

Additionally, a more detailed description of the Behavioral Health Support Specialist (BHSS) role in Washington State would enhance the reader’s understanding of the context and scope of this position. Providing information about the responsibilities and integration of this role within the healthcare system would help readers better interpret the study findings.

The manuscript would also benefit from additional clarification regarding the study setting. Specifically, the authors should indicate the types of clinics or healthcare settings from which participants were recruited.

Finally, it would be helpful for the authors to specify the language(s) in which the interviews were conducted. Given the diversity of the United States population, clarifying whether interviews were conducted only in English or in multiple languages is important. If participants who spoke other languages were excluded, this should be clearly stated and discussed as a potential limitation.

Overall, the manuscript would benefit from revisions to enhance clarity, methodological rigor, and interpretation of the findings. I encourage the authors to address the comments provided to strengthen the manuscript.

**Reviewer #3:**  Thank you for the opportunity to contribute to the review of this manuscript. This study addresses the critical issue of task sharing among (mental) health professionals at a time when many countries are concerned about the shortage of human resources in this field. First, I want to congratulate the authors on the quality of their work. Below, I have made some suggestions for improvement, which I leave to their discretion.

Abstract

The manuscript sometimes refers to “potential patients” and sometimes to “patients.” It is important to harmonize this concept (throughout the manuscript) to comply with what is indicated in the title.

This is intended to be a mixed-methods study, but the abstract does not contain any quantitative results. Please add quantitative findings if possible.

What does BHSS mean? Please avoid acronyms in the abstract.

Introduction

It is stated that task-sharing has been chosen as a strategy to address the shortage of advanced mental health professionals, but there are no statistics to support this claim. Please provide some if they exist.

The manuscript states that the Washington State Department of Health has clarified the BHSS framework and that formative work has been done to define their role. However, it is important to clearly present in this manuscript the tasks shared with BHSS as clinicians. In addition, it is necessary to indicate whether these tasks would be performed in primary care settings or specialized hospitals.

Materials and Methods

It is stated that the study was presented in accordance with COREQ and GRAMMS guidelines, which is excellent. However, the manuscript, which refers potential readers to supplementary files, does not clearly and separately present the various traditional subsections, such as study design, study setting, and data collection procedure. I strongly advise providing the essential information for each of these subsections before referring to the supplementary files.

The “Participants” subsection provides information on both the participants and the sampling procedure. It could be renamed “Participants and Sampling.”

Some information is mixed together in a single subsection, such as the “Participants” subsection, which includes both participants, the data collection period, etc. For clarity, I suggest clearly separating this information into corresponding subsections.

I suggest providing operational definitions of the concepts of “acceptability” and “appropriateness” in the manuscript and then citing the reference, such as Proctor et al. (44), if these are the definitions adopted, instead of simply referring to the reference.

The manuscript describes the descriptive statistical analyses that were performed. However, it provides little or no clear information about the qualitative analyses performed. I suggest clarifying this information.

If the possible responses to the quantitative data collection tool ranged from 1 = Not at all to 4 = Very much and included “I don't know,” this implies that the variables were qualitative (i.e., categorical variables). If this is the case, it is generally accepted that, for this type of variable, frequencies and proportions are estimated and then expressed as percentages. Because the mean and standard deviation were calculated, the authors would do well to explain in the manuscript how they transformed the qualitative variables into quantitative variables to estimate these measures of central tendency and dispersion.

Results

For the description of the participant profile, as the sample size (N = 40) is already mentioned, I suggest simply indicating the percentages when presenting the results and deleting the numbers (n = x), as this gives an impression of repetition.

These “Thirty-eight participants completed the interview with a usable audio-recording. Thirty-nine participants completed the survey.” statements do not constitute participant characteristics. They can be moved to the Methods section.

Please clarify the quantitative findings. Stating “... aligning with moderate to strong interest in the training backgrounds of their mental health providers and moderate to strong support for receiving care from a task-shared mental health provider.” is not very clear to readers.

In Table 1, is it possible to replace the questions asked participants with research variables? This would make these results a little easier to understand.

The text in the “Integration of qualitative and quantitative data” subsection reads like a conclusion. Please present this subsection more clearly so that it reflects the mutual influence of the qualitative and quantitative findings.

Discussion

I suggest elaborating a little more clearly on the strengths, limitations, and study implications and, if possible, making it a separate subsection.

**********

6. PLOS authors have the option to publish the peer review history of their article (what does this mean?). If published, this will include your full peer review and any attached files.

**Do you want your identity to be public for this peer review?**  If you choose “no”, your identity will remain anonymous but your review may still be made public.

For information about this choice, including consent withdrawal, please see our Privacy Policy.

Reviewer #1: No

Reviewer #2: No

Reviewer #3: **Yes:** Erick Mukala Mayoyo

**********

[NOTE: If reviewer comments were submitted as an attachment file, they will be attached to this email and accessible via the submission site. Please log into your account, locate the manuscript record, and check for the action link "View Attachments". If this link does not appear, there are no attachment files.]

-->Figure Resubmissions:

While revising your submission, we strongly recommend that you use PLOS’s NAAS tool (https://ngplosjournals.pagemajik.ai/artanalysis) to test your figure files. NAAS can convert your figure files to the TIFF file type and meet basic requirements (such as print size, resolution), or provide you with a report on issues that do not meet our requirements and that NAAS cannot fix.

After uploading your figures to PLOS’s NAAS tool - https://ngplosjournals.pagemajik.ai/artanalysis, NAAS will process the files provided and display the results in the "Uploaded Files" section of the page as the processing is complete. If the uploaded figures meet our requirements (or NAAS is able to fix the files to meet our requirements), the figure will be marked as "fixed" above. If NAAS is unable to fix the files, a red "failed" label will appear above. When NAAS has confirmed that the figure files meet our requirements, please download the file via the download option, and include these NAAS processed figure files when submitting your revised manuscript.

# Author comment: Author response to Decision Letter 1

**DOI:** 10.1371/journal.pmen.0000606.r002

24 Apr 2026

Attachment Submitted filename: Revision_letter_final.docx

# Peer review: Decision Letter 1

**DOI:** 10.1371/journal.pmen.0000606.r003

**Authors:** Lambert Zixin Li, Lambert Zixin Li

14 May 2026

PMEN-D-26-00055R1

Exploring patient perspectives on a new task-shared behavioral health role in Washington State

PLOS Mental Health

Dear Dr. Rose,

Thank you for submitting your manuscript to PLOS Mental Health. After careful consideration, we feel that it has merit but does not fully meet PLOS Mental Health’s publication criteria as it currently stands. Therefore, we invite you to submit a revised version of the manuscript that addresses the points raised during the review process.

Please submit your revised manuscript by Jun 13 2026 11:59PM. If you will need more time than this to complete your revisions, please reply to this message or contact the journal office at mentalhealth@plos.org. When you're ready to submit your revision, log on to https://www.editorialmanager.com/pmen/ and select the 'Submissions Needing Revision' folder to locate your manuscript file.

Please include the following items when submitting your revised manuscript:

- A letter that responds to each point raised by the editor and reviewer(s). You should upload this letter as a separate file labeled 'Response to Reviewers'.
- A marked-up copy of your manuscript that highlights changes made to the original version. You should upload this as a separate file labeled 'Revised Manuscript with Track Changes'.
- An unmarked version of your revised paper without tracked changes. You should upload this as a separate file labeled 'Manuscript'.

Guidelines for resubmitting your figure files are available below the reviewer comments at the end of this letter.

As the corresponding author, your ORCID iD is verified in the submission system and will appear in the published article. PLOS supports the use of ORCID, and we encourage all coauthors to register for an ORCID iD and use it as well. Please encourage your coauthors to verify their ORCID iD within the submission system before final acceptance, as unverified ORCID iDs will not appear in the published article. *Only*  the individual author can complete the verification step; PLOS staff *cannot*  verify ORCID iDs on behalf of authors.

We look forward to receiving your revised manuscript.

Kind regards,

Lambert Zixin Li, Ph.D.

Academic Editor

PLOS Mental Health

Journal Requirements:

If the reviewer comments include a recommendation to cite specific previously published works, please review and evaluate these publications to determine whether they are relevant and should be cited. There is no requirement to cite these works unless the editor has indicated otherwise.

Please review your reference list to ensure that it is complete and correct. If you have cited papers that have been retracted, please include the rationale for doing so in the manuscript text, or remove these references and replace them with relevant current references. Any changes to the reference list should be mentioned in the rebuttal letter that accompanies your revised manuscript. If you need to cite a retracted article, indicate the article’s retracted status in the References list and also include a citation and full reference for the retraction notice.

Additional Editor Comments (if provided):

Thank you for submitting your revised manuscript. The paper has improved substantially and is now close to being suitable for publication. After considering the latest review and my own assessment, I would like to invite a minor revision to address a few remaining comments raised by one reviewer. Please revise the manuscript accordingly and provide a brief point by point response. I look forward to receiving the revised version.

[Note: HTML markup is below. Please do not edit.]

Reviewers' comments:

Reviewer's Responses to Questions

**Comments to the Author**

1. If the authors have adequately addressed your comments raised in a previous round of review and you feel that this manuscript is now acceptable for publication, you may indicate that here to bypass the “Comments to the Author” section, enter your conflict of interest statement in the “Confidential to Editor” section, and submit your "Accept" recommendation.

Reviewer #2: All comments have been addressed

Reviewer #3: All comments have been addressed

**********

2. Does this manuscript meet PLOS Mental Health’s publication criteria? Is the manuscript technically sound, and do the data support the conclusions? The manuscript must describe methodologically and ethically rigorous research with conclusions that are appropriately drawn based on the data presented.

Reviewer #2: Yes

Reviewer #3: Yes

**********

3. Has the statistical analysis been performed appropriately and rigorously?

Reviewer #2: N/A

Reviewer #3: Yes

**********

4. Have the authors made all data underlying the findings in their manuscript fully available (please refer to the Data Availability Statement at the start of the manuscript PDF file)?

The PLOS Data policy requires authors to make all data underlying the findings described in their manuscript fully available without restriction, with rare exception. The data should be provided as part of the manuscript or its supporting information, or deposited to a public repository. For example, in addition to summary statistics, the data points behind means, medians and variance measures should be available. If there are restrictions on publicly sharing data—e.g. participant privacy or use of data from a third party—those must be specified.

Reviewer #2: No

Reviewer #3: Yes

**********

5. Is the manuscript presented in an intelligible fashion and written in standard English?

PLOS Mental Health does not copyedit accepted manuscripts, so the language in submitted articles must be clear, correct, and unambiguous. Any typographical or grammatical errors should be corrected at revision, so please note any specific errors here.

Reviewer #2: Yes

Reviewer #3: Yes

**********

6. Review Comments to the Author

Please use the space provided to explain your answers to the questions above. You may also include additional comments for the author, including concerns about dual publication, research ethics, or publication ethics. (Please upload your review as an attachment if it exceeds 20,000 characters)

Reviewer #2: Thank you for your thoughtful revisions to the initial version of the manuscript. The updated draft demonstrates meaningful improvement in clarity and organization. I appreciate the effort invested in addressing the earlier feedback.

I noted, however, that the manuscript indicates 40 participants were recruited, whereas the results section reports findings from 38 participants. Please clarify the disposition of the two participants who were not included in the final analysis, including whether they withdrew, were excluded, or had incomplete data. If applicable, providing possible reasons for withdrawal or exclusion would strengthen the methodological transparency of the study.

In addition, although the interview guide has been included as an appendix, it would enhance the Methods section to incorporate a concise summary of the data collection tools and interview procedures. Briefly describing the structure of the interview guide and the major domains explored would improve readability and provide readers with a clearer understanding of the methodological approach without relying solely on the appendix.

Reviewer #3: Thank you for addressing all my concerns. I have nothing further to add. I hope this has helped to improve the quality of the manuscript.

**********

7. PLOS authors have the option to publish the peer review history of their article (what does this mean?). If published, this will include your full peer review and any attached files.

**Do you want your identity to be public for this peer review?**  If you choose “no”, your identity will remain anonymous but your review may still be made public.

For information about this choice, including consent withdrawal, please see our Privacy Policy.

Reviewer #2: No

Reviewer #3: **Yes:** Erick Mukala Mayoyo

**********

[NOTE: If reviewer comments were submitted as an attachment file, they will be attached to this email and accessible via the submission site. Please log into your account, locate the manuscript record, and check for the action link "View Attachments". If this link does not appear, there are no attachment files.]

-->Figure Resubmissions:

While revising your submission, we strongly recommend that you use PLOS’s NAAS tool (https://ngplosjournals.pagemajik.ai/artanalysis) to test your figure files. NAAS can convert your figure files to the TIFF file type and meet basic requirements (such as print size, resolution), or provide you with a report on issues that do not meet our requirements and that NAAS cannot fix.

After uploading your figures to PLOS’s NAAS tool - https://ngplosjournals.pagemajik.ai/artanalysis, NAAS will process the files provided and display the results in the "Uploaded Files" section of the page as the processing is complete. If the uploaded figures meet our requirements (or NAAS is able to fix the files to meet our requirements), the figure will be marked as "fixed" above. If NAAS is unable to fix the files, a red "failed" label will appear above. When NAAS has confirmed that the figure files meet our requirements, please download the file via the download option, and include these NAAS processed figure files when submitting your revised manuscript.

# Author comment: Author response to Decision Letter 2

**DOI:** 10.1371/journal.pmen.0000606.r004

18 May 2026

Attachment Submitted filename: Revision_letter_5.18.docx

# Editor report: Decision Letter 2

**DOI:** 10.1371/journal.pmen.0000606.r005

**Authors:** Lambert Zixin Li, Lambert Zixin Li, Lambert Zixin Li

24 May 2026

Exploring patient perspectives on a new task-shared behavioral health role in Washington State

PMEN-D-26-00055R2

Dear Ms. Rose,

We are pleased to inform you that your manuscript 'Exploring patient perspectives on a new task-shared behavioral health role in Washington State' has been provisionally accepted for publication in PLOS Mental Health.

Before your manuscript can be formally accepted you will need to complete some formatting changes, which you will receive in a follow up email. A member of our team will be in touch with a set of requests.

Please note that your manuscript will not be scheduled for publication until you have made the required changes, so a swift response is appreciated.

IMPORTANT: The editorial review process is now complete. PLOS will only permit corrections to spelling, formatting or significant scientific errors from this point onwards. Requests for major changes, or any which affect the scientific understanding of your work, will cause delays to the publication date of your manuscript.

If your institution or institutions have a press office, please notify them about your upcoming paper to help maximize its impact. If they'll be preparing press materials, please inform our press team as soon as possible -- no later than 48 hours after receiving the formal acceptance. Your manuscript will remain under strict press embargo until 2 pm Eastern Time on the date of publication. For more information, please contact mentalhealth@plos.org.

Thank you again for supporting Open Access publishing; we are looking forward to publishing your work in PLOS Mental Health.

Best regards,

Lambert Zixin Li, Ph.D.

Academic Editor

PLOS Mental Health

***********************************************************

Thank you for submitting your revised manuscript. I have assessed the minor revision and am pleased to inform you that the manuscript is accepted for publication. As the remaining issues were minor, no further external review was required. Thank you for choosing the journal for your work.

Reviewer Comments (if any, and for reference):
